# Prognostic impact of thoracic duct lymph node metastasis in esophageal squamous cell carcinoma

**DOI:** 10.1002/ags3.12432

**Published:** 2021-01-19

**Authors:** Satoru Matsuda, Hirofumi Kawakubo, Hiroya Takeuchi, Shuhei Mayanagi, Tomoyuki Irino, Kazumasa Fukuda, Rieko Nakamura, Norihito Wada, Yuko Kitagawa

**Affiliations:** ^1^ Department of Surgery Keio University School of Medicine Tokyo Japan; ^2^ Department of Surgery Hamamatsu University School of Medicine Hamamatsu Japan

**Keywords:** esophageal cancer, esophageal squamous cell carcinoma, thoracic duct, thoracic duct lymph node metastasis

## Abstract

**Aim:**

We have previously reported the existence of lymph nodes surrounding the thoracic duct ( TDLN) and transthoracic esophagectomy (TTE) with thoracic duct (TD) resection increased the number of lymph nodes (LNs) retrieved. The current study aims to evaluate the prognostic impact of TDLN metastasis in esophageal cancer patients subdivided by its location and comparing the patients’ survival with those with extra‐regional LN metastasis.

**Methods:**

Patients who underwent TTE with TD resection for esophageal squamous cell carcinoma (ESCC) were reviewed. Patients were classified into those with or without TDLN metastasis, and clinicopathological factors were compared between groups. TDLN was further divided into TDLN‐Ut/Mt/Lt based on the location in the mediastinum. The relapse‐free survival (RFS) and overall survival (OS) were compared between groups.

**Results:**

Of 232 patients, TDLN metastasis was observed in 17 (7%). RFS and OS were significantly worse in the TDLN metastasis group. TDLN metastasis was shown to be an independent prognostic factor for RFS and OS in the multivariate analysis. The negative prognostic impact of TDLN metastasis was evident in TDLN‐Mt/Lt. The RFS and OS of patients with TDLN metastasis were almost identical to those with positive LN metastasis in extra‐regional LNs.

**Conclusion:**

TDLN metastasis was proven to be a strong prognostic indicator. Although the TDLN has been included in the classification of regional LN in the current staging systems, it could be independently classified from the current regional LNs. Given that neoadjuvant therapy has been a standard, we might need to introduce adjuvant therapy when TDLN metastasis is observed.

## Graphical abstract

Metastasis at the lymph node surrounding the thoracic duct (TDLN) was proven to be a strong prognostic indicator. Although the TDLN has been included in the classification of regional LN in the current staging systems, it could be separately classified and dealt with as distant metastasis.

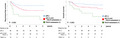

## 1 INTRODUCTION

Esophageal cancer that can metastasize widely from an early stage has been regarded as a devastating disease.^1^, ^2^ While multidisciplinary treatment advances have improved the survival,^3^ esophageal cancer is still the eighth leading cause of cancer‐related deaths in 2018.^4^ Trans‐thoracic esophagectomy (TTE) with lymph node (LN) dissection has been recognized as one of the standard treatments for esophageal cancer, especially in esophageal squamous cell carcinoma (ESCC), where LN metastasis spreads from the cervical to abdominal nodes.^5^ When sentinel LNs were evaluated in ESCC at the mid thoracic esophagus, cervical and abdominal LNs were identified as sentinel LNs in more than 10% of patients.^6^ Therefore, extensive LN dissection such as three‐field lymphadenectomy is recognized as one of the standard treatments for ESCC.^7^, ^8^ The fact that the number of LNs retrieved was previously shown to be a prognostic factor^9^, ^10^ further supported the concept in which radical lymphadenectomy was a vital procedure for the treatment of patients with esophageal cancer.

The thoracic duct (TD) is the main lymphatic root, which originates from the cistern of chyle and ascends along the thoracic descending aorta, flowing at a left venous angle. The pros and cons of TD resection in esophagectomy have been debatable. We have recently shown that minimally invasive TTE with extensive LN dissection along with TD dissection improved survival especially in cStage I esophageal cancer; that was the first study to suggest the survival benefit of TD resection in TTE.^11^ Conversely, two studies reported showing that TD resection could increase the postoperative complication and did not improve the survival.^12^, ^13^ When the oncological outcome of TD resection is investigated, attention needs to be given to the LNs surrounding the TD (TDLN). Previously, Udagawa et al^14^ showed that the TDLN were the nodes in the adipose tissue surrounding the TD and running between the thoracic esophagus and the descending aorta. They indicated that TD resection with dissection of the TDLN should be performed routinely. Following this report, we also demonstrated the existence of TDLN. In Ivor Lewis esophagectomy, which is conducted for adenocarcinoma, Schurink reported TDLN in a cadaver study.^15^ However, there are no studies investigating the prognosis of patients who had metastasis in the TDLN subdivided by its location.

In the present study, we expanded the cohorts of patients with ESCC whose metastatic status of TDLN was pathologically evaluated. The incidence rate of TDLN metastasis was reviewed, and the correlation between TDLN metastasis and survival was analyzed.

## 2 METHODS

### 2.1 Patients and treatment

As previously reported, since June 2013, the TDLN has been examined independently of paratracheal and aortic LNs in our institution.^16^ All patients who underwent TTE with TD resection for esophageal malignancies at the Keio University Hospital between June 2013 and December 2019 were reviewed. We excluded patients with a cT4 tumor, adenocarcinomas, patients that underwent salvage esophagectomy after definitive chemoradiotherapy, and those with R2 resection. As it has been reported that the resection of the TD might influence postoperative fluid retention and liver function,^17^, ^18^ the TD was preserved in patients with liver cirrhosis, kidney, or heart diseases.

Clinical staging was performed with esophagoduodenoscopy, esophagography, and computed tomography, and the 8th edition of the TNM classification established by the Union for International Cancer Control (UICC) was used.^19^ To define the regional and extra‐regional LNs, we referred to the Japanese Classification of Esophageal Cancer 11th edition in which LN was categorized from N1 to N4 based on the location of the primary tumor.^20^, ^21^ Then, N 1‐3 groups were defined as regional LNs in the current analysis. Extra‐regional LNs included upper cervical nodes, common hepatic artery nodes, splenic artery nodes, and infradiaphragmatic nodes. Based on the Japan Clinical Oncology Group (JCOG) 9907 study,^22^ neoadjuvant chemotherapy, using cisplatin and 5‐fluorouracil, was the standard treatment since 2007. A regimen consisting of three drugs (cisplatin, 5‐fluorouracil, and docetaxel) administered three times every 3 weeks was considered for patients who had either border‐line resectable disease or multiple LN metastases at diagnosis.^23^ For those who were diagnosed as cT1N0 and underwent upfront surgery, adjuvant chemotherapy was provided when the patients were found to have LN metastasis in the resected specimen. The present study was approved by the ethics committee of the Keio University School of Medicine.

### 2.2 Surgical procedure, LN station numbers, and surgical outcomes

As a curative surgery, we performed TTE with right thoracotomy and gastric tube reconstruction in the posterior mediastinal route as a standard surgical procedure at our institution. We performed reconstruction using the terminal ileum and right colon when the gastric tube could not be used because of synchronous double cancer of the stomach or previous history of gastrectomy. For a minimally invasive approach, since 2009, we have been conducting transthoracic minimally invasive esophagectomy with a hybrid position‐prone and left lateral decubitus.^24^

In regard to LN dissection, mediastinal LNs with bilateral recurrent nerve LNs and abdominal LNs, including pericardial LNs, and LNs along the lesser curvature and the left gastric artery were routinely dissected. Supraclavicular LNs were also dissected if the primary tumor was located between the upper‐ and mid‐thoracic esophagus. When the LN around the left recurrent nerve was dissected from the aortic arch to the cervical level, the TD and surrounding adipose tissue containing TDLNs was dissected simultaneously. The TD was clipped and divided at the level of the thoracic inlet. In the middle and lower mediastinal approach, after dividing the esophagus and para‐esophageal LNs, the TD and the surrounding adipose tissue were divided from the descending aorta with posterior mediastinal LNs. The TD was then clipped behind the lower thoracic esophagus and resected above the diaphragm. In terms of TDLN, based on the location in the mediastinum, TDLNs were classified into the following three parts: TDLN‐Ut, TDLN in the upper mediastinum; TDLN‐Mt, TDLN in the middle mediastinum; and TDLN‐Lt, TDLN in the lower mediastinum. Especially in terms of the definition of TDLN‐Ut, dissecting the plane including the sympathetic nerve, the left pleura and the aortic arch are identified in the upper mediastinum in the dorsal mobilization of the esophagus as introduced in the surgical procedure in a previous report.^11^ Therefore, the TD and all the surrounding lymphatic/connective tissues are to be mobilized together. The plane, which included the sympathetic nerve behind the left recurrent laryngeal nerve, TD, and other lymphatic/connective tissues, were dissected after the lymphadenectomy surrounding left recurrent laryngeal nerve was done. LNs are occasionally identified and classified as TDLN‐Ut in those tissues.

Postoperative complications were evaluated using the Clavien‐Dindo (CD) classification. In the current study, we investigated the incidence of pneumonia (CD ≥ Grade II), anastomotic leakage (CD ≥ Grade IIIa), chylothorax (CD ≥ Grade IIIa), and postoperative mortality. We analyzed relapse‐free survival (RFS) and overall survival (OS) in patients who underwent TTE before 31 April 2018. The RFS and OS were calculated from the date of surgery to the date of recurrence. Patients were followed up until death or 31 April 2020.

### 2.3 Statistical analysis

We calculated the means and standard deviations, and identified differences using the Student's *t*‐test. We identified differences between categories using the chi‐square test or Fisher's exact test. We produced survival curves using the Kaplan‐Meier survival method. We compared two groups using a two‐sided log‐rank test. We performed all statistical analyses using IBM SPSS Statistics version 28 for Windows (Chicago, IL, USA), and differences were considered significant when *P* < .05.

## 3 RESULTS

### 3.1 Clinicopathological factors of the patients and surgical outcomes between with and without TDLN metastasis

The characteristics of the patients are shown in Table 1. Of 232 patients, 84% were male and the primary tumor was located at the mid‐thoracic esophagus in 118 (51%) patients. Due to supraclavicular LN metastasis, 14 patients (6%) were evaluated as having cM1 disease. The distribution of pre‐treatment clinical stage of 1/2/3/4 was 105 (46%)/35 (15%)/73 (31%)/19 (8%) and postoperative pathological stage of 0/1/2/3/4 was three (1%)/91 (39%)/46 (20%)/58 (25%)/34 (15%), respectively.

**TABLE 1:** Clinicopathological characteristics of the patients

	All patients	TDLN mets (−)	TDLN mets (+)	*P*
	n = 232	n = 215	n = 17	
Age (mean ± SD)	65.5 ± 8.26	65.5 ± 8.32	65.4 ± 7.74	.960
Sex				
Male	194 (84%)	179 (83%)	15 (88%)	.593
Female	38 (16%)	36 (17%)	2 (12%)	
Location				
Ut	35 (15%)	33 (15%)	2 (15%)	.787
Mt	118 (51%)	108 (50%)	10 (51%)	
Lt	79 (34%)	74 (35%)	5 (34%)	
NAC or NACRT				
No	95 (41%)	90 (31%)	5 (29%)	.315
Yes	137 (59%)	125 (69%)	12 (71%)	
Depth of invasion				
cT1	115 (50%)	112 (52%)	3 (18%)	.002
cT2	33 (14%)	32 (15%)	1 (6%)	
cT3	81 (35%)	69 (32%)	12 (70%)	
cT4a	3 (1%)	2 (1%)	1 (6%)	
Lymph node metastasis				
cN0	112 (48%)	106 (49%)	6 (35%)	.014
cN1	70 (30%)	67 (31%)	3 (18%)	
cN2	48 (21%)	41 (19%)	7 (41%)	
cN3	2 (1%)	1 (1%)	1 (6%)	
Distant metastasis				
cM0	218 (94%)	202 (94%)	16 (94%)	.978
cM1 (supraclavicular LN)	14 (6%)	13 (6%)	1 (6%)	
Clinical stage				
cStage I	105 (46%)	102 (48%)	3 (18%)	.086
cStage II	35 (15%)	32 (15%)	3 (18%)	
cStage III	73 (31%)	65 (30%)	8 (47%)	
cStage IV	19 (8%)	16 (7%)	3 (17%)	
Pathological depth of invasion				
pT0	6 (3%)	6 (2%)	0 (0%)	.001
pT1	131 (56%)	127 (59%)	4 (24%)	
pT2	20 (9%)	20 (9%)	0 (0%)	
pT3	73 (31%)	61 (28%)	12 (71%)	
pT4a	2 (1%)	1 (1%)	1 (5%)	
Pathological lymph node metastasis				
pN0	120 (52%)	120 (56%)	0 (0%)	<.001
pN1	60 (26%)	59 (27%)	1 (6%)	
pN2	38 (16%)	29 (14%)	9 (53%)	
pN3	14 (6%)	7 (3%)	7 (41%)	
Pathological distant metastasis				
pM0	208 (90%)	195 (91%)	13 (77%)	.064
pM1 (supraclavicular LN)	24 (10%)	20 (9%)	4 (23%)	
Pathological stage				
pStage 0	3 (1%)	3 (1%)	0 (0%)	<.001
pStage I	91 (39%)	91 (42%)	0 (0%)	
pStage II	46 (20%)	46 (22%)	0 (0%)	
pStage III	58 (25%)	50 (23%)	8 (47%)	
pStage IV	34 (15%)	25 (12%)	9 (53%)	
Histological grade				
pGrade 0	18 (14%)	15 (13%)	3 (25%)	.415
pGrade 1a	75 (56%)	67 (55%)	8 (67%)	
pGrade 1b	18 (14%)	18 (15%)	0 (0%)	
pGrade 2	16 (12%)	15 (13%)	1 (8%)	
pGrade 3	5 (4%)	5 (4%)	0 (0%)	

Abbreviations: LN, lymph node; Lt, lower thoracic esophagus to the esophagogastric junction; Mets, metastasis; Mt, mid‐thoracic esophagus; NAC, neoadjuvant chemotherapy; NACRT, neoadjuvant chemoradiotherapy; TDLN, LN around thoracic duct; Ut, upper thoracic esophagus.

TDLN metastasis was observed in 17 (7%) patients. When the characteristics and clinicopathological factors of the patients were compared between the patients with and without TDLN metastasis, there was no significant difference in age, sex, location, and the administration of neoadjuvant therapy. Patients with TDLN metastasis exhibited significantly advanced stage. Fifteen out of 16 patients showed pN2 or pN3 disease, indicating that TDLN metastasis occurred as one of multiple LN metastases.

In terms of the surgical approaches and short‐term outcomes (Table S1), there was no significant difference in the amount of intraoperative bleeding, while operative time was significantly shorter in patients with TDLN metastasis. The percentages of thoracoscopic and robotic esophagectomy were significantly higher in the TDLN negative group. The incidence rate of postoperative anastomotic leakage (≥ CD III) and pneumonia (≥ CD II) was 10% and 14%, respectively, and there was no significant difference between patients with and without TDLN metastasis. There was a postoperative mortality in a patient without TDLN metastasis.

### 3.2 Distribution of TDLN metastasis

**FIGURE 1:**
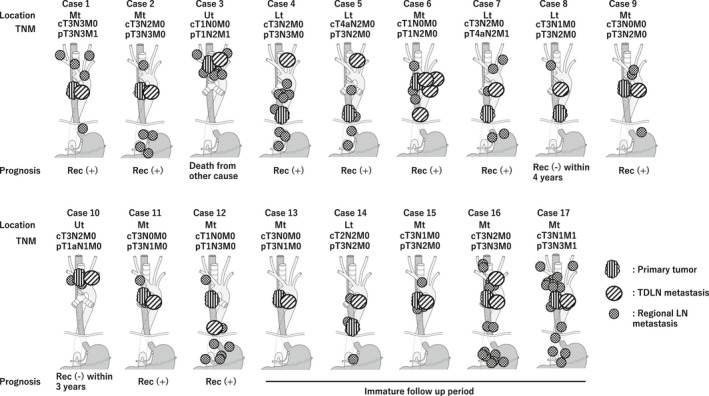
The distribution of metastasis in the thoracic duct lymph node. All patients who were diagnosed as positive metastasis in the lymph nodes around the thoracic duct were described along with the TNM staging

The distribution of TDLN metastasis is described in Figure 1. TDLN‐Ut/‐Mt/‐Lt metastasis was observed in 5 (29%)/12 (71%)/2 (12%) patients, respectively, indicating that TDLN metastasis occurred frequently at the mid‐thoracic esophagus. Fifteen patients showed TDLN metastasis at a single LN, while one patient had metastases both in TDLN‐Mt and TDLN‐Lt, while another had metastases in TDLN‐Ut and Mt. When the correlation between TDLN metastasis and other lesions was investigated, 16 out of 17 patients (94%) showed a primary tumor or LN metastasis in the non‐TDLN group at the same level or caudal side to the metastatic TDLNs.

### 3.3 TDLN metastasis, survival, and distribution of postoperative recurrence

**FIGURE 2:**
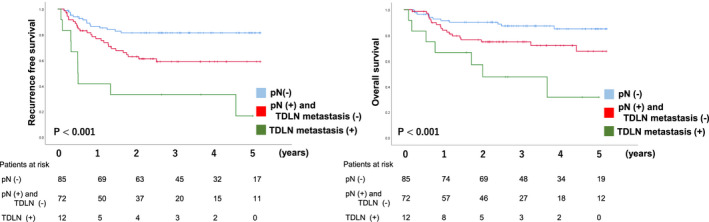
The metastasis in the thoracic duct lymph node and survival

**FIGURE 3:**
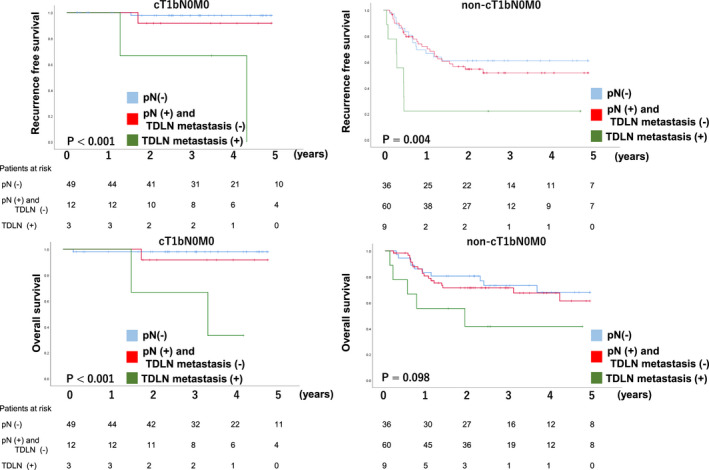
The metastasis in the thoracic duct lymph node and survival stratified into cT1bN0M0 and non‐cT1bN0M0

**FIGURE 4:**
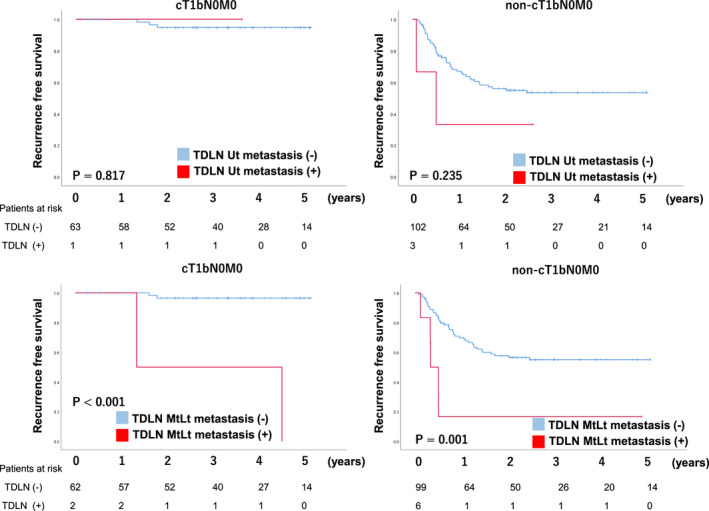
The correlation between the location of the thoracic duct lymph node metastasis and relapse‐free survival

Survival analyses were performed in patients who underwent TTE between 2013 and April 2018. The median follow‐up period was 1016 days. RFS and OS were significantly worse in the TDLN metastasis group, 5‐year RFS and 5‐year OS of the pN (−)/pN (+) and TDLN metastasis (−)/TDLN (+) were 81%/59%/17% (*P* < .01) and 85%/68%/32% (*P* < .01) respectively (Figure 2). Under the stratification based on pretreatment stage, while the incidence rate of TDLN metastasis was relatively rare in cT1bN0M0, in which surgical resection without preoperative therapy is a standard, patients with TDLN metastasis showed significantly shorter RFS and OS (Figure 3). Of 64 patients with cT1bN0, 16 patients (25%) were diagnosed to have LN metastasis in the resected specimen and nine out of 16 patients (56%) received adjuvant chemotherapy. In advanced cancer (non‐cT1bN0M0), the 5‐year RFS rate of patients with TDLN metastasis was 22%, which was significantly lower than the 56% in those without TDLN metastasis. When we classified patients with TDLN metastasis into TDLN‐Ut group and TDLN‐Mt/Lt group, the negative prognostic impact of TDLN metastasis was more evident in TDLN‐Mt/Lt (Figure 4). The only patients who survived without recurrence exhibited remarkable pathological response after neoadjuvant chemotherapy.

To evaluate whether the negative impact of TDLN metastasis on survival was independent of pathological factors, we conducted a multivariate survival analysis. In the analysis using TDLN metastasis, pStage, and histological grade as covariates, TDLN metastasis was shown to be an independent prognostic factor for RFS (Hazard ratio [HR], 4.27; *P* = .003) and OS (HR, 3.35; *P* = .021) (Table 2).

**TABLE 2:** Clinicopathological factors, TDLN metastasis, and survival: Uni‐ and multivariate analysis

n = 169	Univariate analysis			Multivariate analysis		
	HR	*P*	95% CI	HR	*P*	95% CI
Recurrence‐free survival						
TDLN metastasis						
None	Ref			Ref		
Present	4.32	<.001	2.09‐8.89	4.27	.003	1.62‐11.23
pStage						
0/I	Ref			Ref		
II	8.35	<.001	2.75‐25.40	2.84	.107	0.80‐10.12
III/IV	14.16	<.001	5.05‐39.76	2.67	.115	0.79‐9.05
pGrade						
0/1a	Ref			Ref		
1b/2/3	0.33	.005	0.16‐0.71	0.37	.017	0.17‐0.84
Overall survival						
TDLN metastasis						
None	Ref			Ref		
Present	3.90	.001	1.71‐8.90	3.35	.021	1.20‐9.36
pStage						
0/I	Ref			Ref		
II	6.38	.002	2.03‐20.06	2.29	.304	0.47‐11.16
III/IV	9.39	<.001	3.29‐26.81	2.59	.212	0.58‐11.52
pGrade						
0/1a	Ref			Ref		
1b/2/3	0.24	.008	0.08‐0.69	0.31	.034	0.11‐0.92

Abbreviations: LN, lymph node; TDLN, LN around thoracic duct.

**FIGURE 5:**
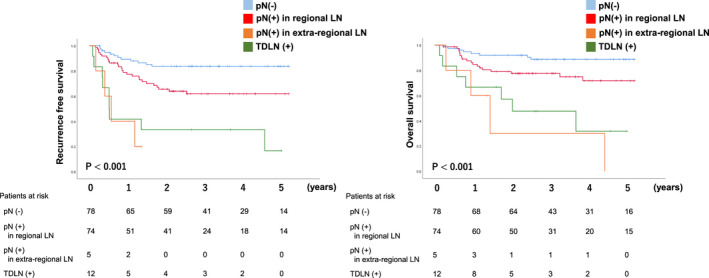
The survival of thoracic duct lymph node metastasis compared to regional and extra‐regional LNs metastasis. To define the regional and extra‐regional LNs, we followed the Japanese Classification Esophageal Cancer 11th edition

Following the Japanese Classification of Esophageal Cancer, we compared the survival of patients with TDLN metastasis to that of those who had metastasis in the regional and extra‐regional LNs. Extra‐regional LNs included upper cervical nodes, common hepatic artery nodes, splenic artery nodes, and infradiaphragmatic nodes. The RFS and OS of patients with TDLN metastasis was almost identical to those with positive LN metastasis in extra‐regional LNs (Figure 5).

When the distribution of initial postoperative recurrence was evaluated (Table S2), the incidence of metastasis at the mediastinal LN (TDLN metastasis [−]/[+], 20 [13%]/4 [33%]; *P* = .049) and distant organs (TDLN metastasis [−]/[+], 16 [9%]/4 [33%]; *P* = .017) were significantly higher in TDLN metastasis group, while there was no difference in other sites between groups.

## 4 DISCUSSION

The current study successfully validated our previous findings that there was the existence of LN metastasis around the TD in ESCC.^16^ Subsequently, we further confirmed that TDLN metastasis occurred in the highly advanced stage diseases and the patients with TDLN metastasis has multiple LN metastases in various locations. None of the study participants had solitary LN metastasis in TDLN without non‐TDLN metastasis, indicating that the TDLN might not receive the direct lymphatic flow from primary tumor, as sentinel LNs in ESCC do. In the survival analyses, nine out of 12 patients (75%) in whom TDLN metastasis was observed experienced postoperative recurrence. This led us to the conclusion that the prognosis in patients with TDLN metastasis is extremely poor. Through the multivariate analysis, which took into account the tumor stage, we were convinced that the TDLN metastasis was an independent prognostic factor for RFS and OS. Consequently, TDLN metastasis was found to be a strong prognostic indicator in ESCC. Although more sample collection is required, this is the first study which has found a direct association between TDLN metastases for each location and dismal prognosis in esophageal cancer.

In contrast to the value of TDLN metastasis as a prognostic indicator, the efficacy of lymphadenectomy of TDLN with TD resection might not be remarkable. Based on the current study, which was the first study evaluating the prognosis of patients with TDLN metastasis for each location, the incidence rate of TDLN metastasis was relatively low, and its prognosis was proven to be extremely poor. In a previous study, to evaluate the prognostic impact for each LN station, the efficacy index, which takes into account the incidence rate and prognosis of the patients with metastasis in the lesion, was introduced.^25^ When efficacy index is calculated for the TDLN, the index of TDLN could be low because of its low incidence rate and high mortality. Furthermore, the survival in patients with TDLN metastasis was almost identical to those with extra‐regional LN metastasis that was recognized as distant metastasis (Figure 5). Although the TDLN has been included in the regional LN classification, it could be independently classified from the current regional LNs and considered as a strong negative prognostic indicator. On the other hand, in addition to the eradication of the TDLN, there are certainly several mechanisms in which TD resection contributes to the improvement of survival. When TD is resected in TTE, the adipose tissue surrounding the esophagus that could include cancer cells can be removed simultaneously, which might increase the radicality of surgical resection. Furthermore, as we reported, TD resection would increase the number of LNs not only in TDLNs, but LNs around the recurrent laryngeal nerves through extensive LN dissection.^16^ These multiple roles of TD resection consequently confirm the findings of our previous report, that the extensive LN dissection with TD resection improved the survival especially in cStage I ESCC where surgical resection is the standard of care.^11^ On the other hand, no previous study has demonstrated the survival benefit of TD resection in advanced‐stage disease. A prospective randomized trial is necessary to conclude the current debate.

In order to improve the prognosis in patients with TDLN metastasis, adjuvant therapy can be a therapeutic option because patients with TDLN metastasis are to be identified after surgery. A network meta‐analysis reviewed patients who received neoadjuvant therapy. Consequently, those who received adjuvant therapy were compared with those without postoperative treatment.^26^ The survival benefit of adjuvant therapy was significantly confirmed by propensity score matching. A randomized control trial comparing perioperative chemotherapy with preoperative therapy demonstrated that perioperative chemotherapy, in which adjuvant chemotherapy was provided after surgery, showed a significantly longer overall survival for ESCC.^27^ The efficacy of adding adjuvant chemotherapy, even after neoadjuvant treatment, was also confirmed in a phase‐II trial from another cohort.^28^ Therefore, adjuvant therapy can be considered for those with TDLN metastasis.

It is worth noting that the survival impact of TDLN metastasis was evaluated for each location (TDLN‐Ut/Mt/Lt) in the current study. In 12 out of 17 patients (71%), TDLN metastasis was observed along with the mid‐thoracic esophagus. Since the TD runs closely with the esophagus around the azygos vein, the lymphatic network might be abundant and tight around this area. Regarding the anatomical correlation between TDLN metastasis and other metastatic lesions, we previously reported that the primary tumor or LN metastasis in non‐TDLN located at the same level or caudal side to the metastatic TDLNs, reporting cases 1 to 8 without data of prognosis.^16^ We rigidly maintained that working hypothesis in case 9 to 17, which were newly added in the current study. The current result suggested that the lymphatic route along the TD, which runs from the caudal to cranial side, seeds the cancer cells to the TDLN. Hence, this might not be the direct lymphatic route from regional LN to the TDLN. In terms of the survival advantages of TDLN for each location, patients who had metastasis at TDLN‐Mt/Lt showed significantly worse prognosis. On the other hand, the RFS of TDLN‐Ut after surgery was not as poor as TDLN‐Mt/Lt, although the incidence rate of metastasis in TDLN‐Ut was lower than that in TDLN‐Mt/Lt. Given that the distance between the esophagus and TD is slightly closer in the upper thorax than the mid and lower mediastinum, the survival benefit of TDLN might be maintained at TDLN‐Ut.

As always, several drawbacks to the resection of the TD need to be considered. TD ligation was reported to induce retroperitoneal fluid retention and lead to intravenous volume loss after surgery.^18^, ^29^ In terms of hepatic damage, Guler et al demonstrated that TD ligation had a negative effect on the liver in a canine model of peritonitis, which was induced by the exposure of liver to the endotoxin.^17^ Aiko et al reported that TD resection affected the fluid balance and minimized the clinical benefit of enteral feeding after esophagectomy.^29^ Furthermore, two papers from Japan reported a negative impact of TD resection in the postsurgical state. Yoshida et al described that TD resection would increase the incidence of pulmonary comorbidity.^12^, ^13^ Since the anticipated benefits need to outweigh the potential risks, prospective studies that validate these previous results are warranted.

The present study was limited by its retrospective nature. However, consecutive patients who underwent TTE were reviewed and all patients with ESCC who underwent TTE between June 2013 and July 2020 were included in this analysis, leading to reduced selection bias. Regarding survival analysis, the follow‐up is still immature with respect to the study population. Therefore, the cutoff was set at April 2018 because this study intended to include patients who were followed up for >1 year. As RFS was mainly used in the current analysis, the difference in survival was fairly evaluated.

In conclusion, TDLN metastasis was proven to be a strong prognostic indicator in ESCC. Although the TDLN has been included in the regional LN classification in both the current systems, it could be independently classified from the current regional LNs. Given that neoadjuvant therapy has been a standard, we might need to introduce the adjuvant therapy when TDLN metastasis is observed. A prospective comparative trial is necessary for conclusions to be arrived at concerning the survival advantage of TD resection in ESCC.

## DISCLOSURE

Funding: Yuko Kitagawa ‐ Relevant financial activities outside the submitted work: lecture Fees ‐ Chugai Pharmaceutical Co. Ltd., Taiho Pharmaceutical Co. Ltd., Asahi Kasei Pharma Corporation, Otsuka Pharmaceutical Factory Inc., Shionogi & Co. Ltd., Nippon Covidien Inc.; grants ‐ Chugai Pharmaceutical Co. Ltd., Taiho Pharmaceutical Co., Ltd., Yakult Honsha Co. Ltd., Asahikasei Co. Ltd., Otsuka Pharmaceutical Co. Ltd., Takeda Pharmaceutical Co. Ltd., Ono Pharmaceutical Co. Ltd., Tsumura & Co., Kyouwa Hakkou Kirin Co. Ltd., Dainippon Sumitomo Pharma Co. Ltd., EA Pharma Co. Ltd., Astellas Pharma Inc., Toyama Chemical Co. Ltd., Medicon Inc., Kaken Pharmaceutical Co. Ltd., Eisai Co. Ltd., Otsuka Pharmaceutical Factory Inc., Teijin Pharma Ltd., Nihon Pharmaceutical Co. Ltd., Nippon Covidien Inc.; endowed chair ‐ Chugai Pharmaceutical Co. Ltd., Taiho Pharmaceutical Co. Ltd.

## Supporting information

Table S1‐S2
