# Supplementary material for: Prognostic impact of thoracic duct lymph node metastasis in esophageal squamous cell carcinoma
Source: Ann Gastroenterol Surg. 2021 Jan 19;5(3):321–30. doi: 10.1002/ags3.12432 (PMC8164460; doi:10.1002/ags3.12432)
Supplement: Supplementary file 1 — Table S1‐S2 [file AGS3-5-321-s001.docx]

| **Supplementary table 1.** Surgical approaches, outcomes and postoperative complications | | | | | | |
| --- | --- | --- | --- | --- | --- | --- |
|  |  | All patients  n = 232 |  | TDLN mets (-)  n = 215 | TDLN mets (+)  n = 17 | P |
| Surgical approach  Open  Thoracoscopy  Robot |  | 16 (7%)  204 (88%)  12 (5%) |  | 11 (5%)  192 (89%)  12 (6%) | 5 (30%)  12 (70%)  0 (0%) | 0.001 |
| Operative time (min, mean ± SD) |  | 495.7 ± 67.1 |  | 497.6 ± 67.3 | 471.6 ± 60.8 | 0.123 |
| Blood (g, mean ± SD) |  | 200.5 ± 347.1 |  | 198.7 ± 354.2 | 224.2 ± 246.3 | 0.771 |
| Postoperative complication  Anastomotic leakage (>CDIII)  Pneumonia(>CDII)  Chylothorax(>CDIII)  RLN palsy (>CDIII) |  | 23 (10%)  33 (14%)  6 (2.6%)  20 (8.7%) |  | 20 (9%)  29 (14%)  6 (2.8%)  20 (9.3%) | 3 (18%)  4 (24%)  0 (0%)  0 (0%) | 0.268  0.254  0.485  0.188 |
| Mortality within 90 days |  | 1 (0.5%) |  | 1 (0.4%) | 0 (%) | 0.778 |
| LN, lymph node; TDLN, LN around thoracic duct; Mets, metastasis; CD, Clavien dindo classification; RLN, recurrent laryngeal nerve | | | | | | |

| **Supplementary table 2.** Distribution of initial recurrence and TDLN metastasis | | | | | | |
| --- | --- | --- | --- | --- | --- | --- |
|  |  | All patients  n = 169 |  | TDLN mets (-)  n = 157 | TDLN mets (+)  n = 12 | P |
| Number of patients with recurrence |  | 50 (30%) |  | 42 (27%) | 8 (67%) | < 0.001 |
| Site of recurrence  Cervical LN  Mediastinal LN  Abdominal LN  Local (anastomotic site)  Pleura  Distant (Liver, lung, bone, brain, kidney etc.) |  | 8 (5%)  24 (14%)  11 (7%)  3 (2%)  8 (5%)  20 (12%) |  | 7 (5%)  20 (13%)  10 (6%)  3 (2%)  8 (5%)  16 (9%) | 1 (8%)  4 (33%)  1 (8%)  0 (0%)  0 (0%)  4 (33%) | 0.452  0.049  0.790  0.629  0.423  0.017 |
| LN, lymph node; TDLN, LN around thoracic duct | | | | | | |
